# Infants Receive More Care by Harassing Matings in a Multi-Level Primate Society

**DOI:** 10.3390/biology14111571

**Published:** 2025-11-09

**Authors:** Fang-Jun Cao, James R. Anderson, Wei-Wei Fu, Ni-Na Gou, Hui Feng, Xiao-Ning Chen, Li-Na Su, Shu-Jun He, Cheng Fang, Lu Wang, Shan-Shan Sun, Min Mao, Kai-Feng Wang, Bin Yang

**Affiliations:** 1 Shaanxi Key Laboratory of Qinling Ecological Security, Shaanxi Key Laboratory for Animal Conservation, Shaanxi Institute of Zoology, Xi’an 710032, China; 2 Department of Psychology, Graduate School of Letters, and Wildlife Research Center, Kyoto University, Kyoto 606-8501, Japan; 3 Department of Hotel Catering and Business Administration, Technological and Higher Education Institute of Hong Kong, Hong Kong 999077, China

**Keywords:** *Rhinopithecus roxellana*, infant harassment, sexual behavior, parent–offspring conflict, maternal care, behavioral strategy

## Abstract

To better understand the motivation underlying harassment of adult matings by infants, we studied infant harassment behaviors in a multilevel social primate, wild (provisioned) Sichuan snub-nosed monkeys (*Rhinopithecus roxellana*). Two harassment patterns were identified: care-seeking and sociable. Infants showed more frequent harassment in response to sexual activities of their mothers than non-mothers, with more care-seeking harassment directed to their mothers. The responses of mothers and non-mothers to infant harassment were different, with the former showing increased caretaking of their infants. Infants harassing non-mothers were more likely to receive aggression from the latter than infants harassing their own mothers. Overall, the more frequently infants harassed matings, the more care they received and the more attacks they received. We conclude that in Sichuan snub-nosed monkeys, infant harassment of their mother’s sexual activity is an effective behavioral tactic to receive more maternal care. It can therefore be interpreted as indirect support for the parent–offspring conflict hypothesis.

## Simple Summary

The aims of this study were to assess the motivation underlying harassment of adult matings by infants in a multilevel social primate, namely wild (provisioned) Sichuan snub-nosed monkeys, and to describe and quantify the reactions of harassed adults to infant harassment. Infants more frequently harassed sexual activities of their mothers than non-mothers, leading to them receiving more care from their mothers; non-mothers were more likely to respond to infant harassment with aggression. These findings can be taken as providing indirect support for parent–infant conflict theory in as much as infant harassment of their mother’s sexual activity was effective in securing more maternal care. Future work on parent–infant conflict should incorporate behavioral, physiological, and reproductive measures, including pregnancy onset and inter-birth intervals.

## 1. Introduction

Harassment of matings refers to any form of harassment or interference by an individual during other individuals’ sexual activity [1,2,3]. It has been recorded in many species of animals, including more than 30 primate species [4,5,6,7,8,9]. Harassment behaviors may be mild or agonistic. Agonistic forms are mostly shown by adult males and include grabbing, pulling, slapping, hitting, and even biting, which often result in termination of the target’s sexual behavior [7,10]. Milder forms of harassment are mainly shown by immature individuals and adult females and include approaching, touching, grooming, sniffing, or even playing with the target individual [10,11,12,13,14,15]; these behaviors less frequently result in termination of copulation [7,10,12]. In its various forms, harassment may serve different functions across species and across age–sex classes within the same species [10,12]. Although infants of several primate species are reported to harass mating couples [5,7,16,17], this has been less studied than harassment by adults [5,18].

Harassment of matings by immature Sichuan snub-nosed monkeys has been described in captivity [19] and the wild [20,21]. In its agonistic form, an immature may vocalize loudly and pull at one of the mating pair. If their mother is mating, they may climb onto her back or force themselves onto her frontal torso occasionally, thus terminating the copulation. Previous studies have generally overlooked the motivation behind harassment by infants. Here, we quantitatively analyze year-round harassment of sexual activity by infants in wild Sichuan snub-nosed monkeys and explore the motives, associated risks, and benefits of such behavior. For discussion, we make limited reference to parent–offspring conflict. Parents and offspring have conflicting interests when it comes to maximizing inclusive fitness [16]. The hypothesis suggests that, in terms of immediate benefits, harassment of its mother’s mating might enable the infant harasser to receive direct care from the mother. In the longer term, the ultimate benefit is the delay of the mother’s next conception and hence the arrival of a future competitor, i.e., a full- or half-brother or sister. An example of this delaying effect was reported in long-tailed macaques (*Macaca fascicularis*), where infants’ interference in their mothers’ matings resulted in retarded conception [22]. More long-term behavioral and fertility data are required to confirm the ultimate benefits of mother-infant conflict-related behaviors. Here, we focused on largely neglected short-term benefits and risks associated with infant harassment behaviors. We hypothesized that infants would harass their mothers’ sexual behaviors more than those of other females, and that infant harassment was a strategy to strengthen maternal care. Specifically, we predicted that infants would gain more care by harassing their mothers’ copulations than the copulations of other females, and that overall receipt of maternal care would be positively correlated with infant harassment behaviors.

## 2. Materials and Methods

### 2.1. Study Site and Species

We studied infant harassment of matings in the Sichuan snub-nosed monkey (*Rhinopithecus roxellana*), an endangered Asian colobine monkey living in temperate forests of mountain plateaux at an altitude of 1500 to 3400 m in central and southwestern China [23,24]. The fundamental element of social organization in this species is the one-male unit—OMU [25]. Multiple independent OMUs often live together, forming a cohesive breeding band [25,26,27,28]. Several OMUs, each containing one resident male, several adult and subadult females, and immature offspring, share the same home range [26]. Although the monkeys have a mating season and birth season, from September to December and March to May, respectively [20,29], sexual and mating behaviors occur throughout the year, with copulations gradually becoming more frequent from August; harassment of matings can also occur throughout the year. Sexual behavior and harassment can also occur both within the OMU and outside the OMU, although harassment by infants mostly occurs within the OMU [21].

The study site is located in Foping County, Shaanxi Province, central China, near the Qinling village, Guanyin Mountain Nature Reserve (107°51′–108°01′ E, 33°35′–33°45′ N) [24,28]. In order to obtain better observation conditions, provisioning was started on 20 April 2010. Research assistants scatter 3.5 kg of corn and 20 kg of sliced apples and radishes throughout the valley at fixed locations at 09:00, 12:00, and 15:00 every day during the observation period. To reduce the influence of food on the monkeys’ behavior, the amount of food provided is fixed; the monkeys obtain most of their food through natural foraging. At the same time, the provisioned food is scattered to minimize feeding competition. Furthermore, based on observations of the monkey groups that moved away from the feeding site [21] and the behavior of the monkey groups in the absence of food supply, we are confident that it has minimal effects on sexual and infant harassment behaviors. The group is highly accustomed to the presence of the observers and tolerates observer distances of 2 m to 50 m, at which we can easily identify individuals based on sex, body shape, fur color, and any physical abnormalities [27,28]. The monkeys were divided into four age/sex categories: adult male and adult female (5 years or older), juvenile (1 to 5 years old), and infant (under 1 year old) [26].

### 2.2. Data Collection

The study group has been observed since December 2009. The quantitative data used for the present study were collected on eight to nine OMUs (70–90 individuals) from February 2015 to May 2016 (312 days, 1872 h) and from February 2021 to May 2022 (318 days, 1908 h), ensuring that each observed infant was studied from birth to one year of age and that harassment of matings was observed during both mating and non-mating seasons. Focusing on OMUs containing three to seven adult females, 24 infants were studied (12 males, 12 females). We used a combination of focal animal sampling [30] and all-occurrence records [31,32] for infant harassment during daily 6 h observation sessions. The definitions of behaviors recorded in this study (harassment, solicitation, mating) were the same as in [21]. Following initial practice and discussion to ensure standardization, we recorded all instances of harassment by infants during focal sessions, as well as the identities of the harassed females and their kinship with the infants. We distinguished two forms of harassment, namely “care-seeking” (screaming, forcing onto the female’s frontal torso, and suckling) and “sociable” (a calmer approach, grooming, gentle touching, and playing with the female). Regarding touching, we observed no direct attempts by harassing infants to directly access the nipple. We also recorded the response of the harassed individuals: unresponsiveness (ignoring the harassment), agonistic (threatening or hitting the harasser), or care (grooming, carrying, or nursing the harasser) occurring during or within 30 sec of the end of harassment. Adult females who were harassed were classified as either the infant’s mother or a non-mother. Non-mothers included females either genetically related or unrelated to the mother; however, due to the small sample sizes, this distinction was not retained in the analysis.

### 2.3. Statistical Analysis

Statistical analyses were performed using SAS software V. 9.1.3 and GraphPad Prism 9.0. Normality was assessed using the Shapiro–Wilk test. Categorical variables were compared with chi-squared tests and Fisher’s exact test. To analyze influencing factors, A generalized linear mixed model (GLMM) was fitted with an identity link function, and the response variable distribution is the normal distribution, with infant ID (infant IDs are determined by year, OMU, mother, and individual identification) as a random factor. We used GLMM to analyze factors influencing the frequency of infants’ harassment (care-seeking and sociable), infant sex (male and female), and kinship (mother and non-mother) as fixed factors and infant ID (*n* = 24 and 48 events) as a random factor. We used GLMM to analyze factors influencing the frequency of infants’ harassment of matings by mothers and by non-mothers, with the same fixed factors, and infant ID (*n* = 24 and 48 events) as a random factor. We used GLMM to analyze factors influencing the frequency of receiving care and of receiving aggression, with infant sex (male and female), kinship (mother and non-mother), and infant harassment frequency as fixed factors, and infant ID (*n* = 24 and 48 events) as a random factor. We used GLMM to analyze factors influencing the frequencies of mothers’ responses and non-mothers’ responses, with infant sex (male and female), response type (care and aggression), and infant harassment frequency as fixed factors, and infant ID (*n* = 24 and 48 events) as a random factor. Any effect at *p* < 0.05 was considered statistically significant.

### 2.4. Ethics Statement

All studies were approved by the animal care committees of the Wildlife Protection Society of Shaanxi Province, China, and were conducted in accordance with the stated research procedures. All research reported in this article follows the regulatory requirements of Guanyinshan Nature Reserve, China, and the International Primate Society’s guidelines on the ethical treatment of primates. The study was conducted entirely using noninvasive methods unlikely to have any impact on the welfare of the monkeys participating in the study.

## 3. Results

**Figure 1:**
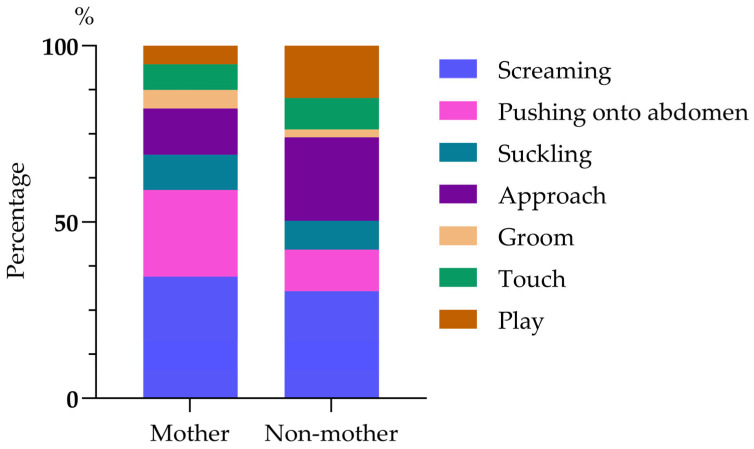
A composition diagram of mating harassment behavior patterns in an infant.

A total of 326 infant harassments were recorded, i.e., on average 1 harassment episode per 11.6 h of observation. Most were of the care-seeking type (61.35%, *n* = 200), with screaming occurring in 32.82% (*n* = 107), pushing onto the torso in 19.33% (*n* = 63), and suckling in 9.20% (*n* = 30). The remaining 126 instances of harassment (38.65%) were of the sociable type, including approach (17.48%, *n* = 57), groom (3.99%, *n* = 13), simple touch (7.98%, *n* = 26), and play (9.20%, *n* = 30, 30/326). Most infant harassment was directed at the infants’ own mother (58.59%, *n* = 191) and included screaming (34.55%, *n* = 66, 66/191), pushing onto the torso (24.61%, *n* = 47), suckling (9.95%, *n* = 19), approaching (13.09%, *n* = 25), grooming (5.24%, *n* = 10), touching (7.33%, *n* = 14) and playing (5.24%, *n* = 10). When infants harassed non-mothers (41.41%, n = 135), they screamed (30.37%, *n* = 41), pushed onto torso (11.85%, *n* = 16), attempted to suckle (8.15%, *n* = 11), approached (23.70%, *n* = 32), groomed (2.22%, *n* = 3), touched (8.89%, *n* = 12), and played (14.81%, *n* = 20). There were significant differences in the behaviors shown by infants when harassing their mothers and non-mothers (chi-squared test: χ^2^(6) = 22.385, *p* < 0.01; Figure 1).

GLMM showed no interaction between infant sex and kinship (*p* > 0.05; Table 1) concerning the frequency of harassment. Infant sex had no overall influence (*p* > 0.05; Table 1), whereas kinship had a significant impact, with harassment more frequently targeting mothers than non-mothers (*p* < 0.05; Table 1). Kinship affected the frequency of care-seeking harassment, which was directed more toward mothers than non-mothers (*p* < 0.05; Table 1). However, there was no significant difference in the frequency of sociable harassment by infants toward their mothers and non-mothers (*p* > 0.05; Table 1).

GLMM showed no overall interaction between infant’s sex and harassment type (*p* > 0.05; Table 2). There was no overall influence of the infant’s sex (*p* > 0.05; Table 2), while mother-directed care-seeking harassment was significantly more frequent than sociable harassment (*p* < 0.05; Table 2). Care-seeking and sociable harassment of non-mothers were not significantly different in frequency (*p* > 0.05; Table 2).

**Figure 2:**
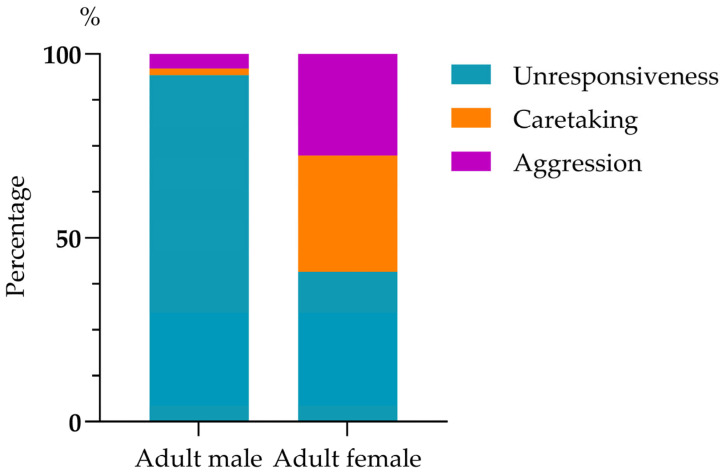
Responses of the harassed adult males and adult females.

**Figure 3:**
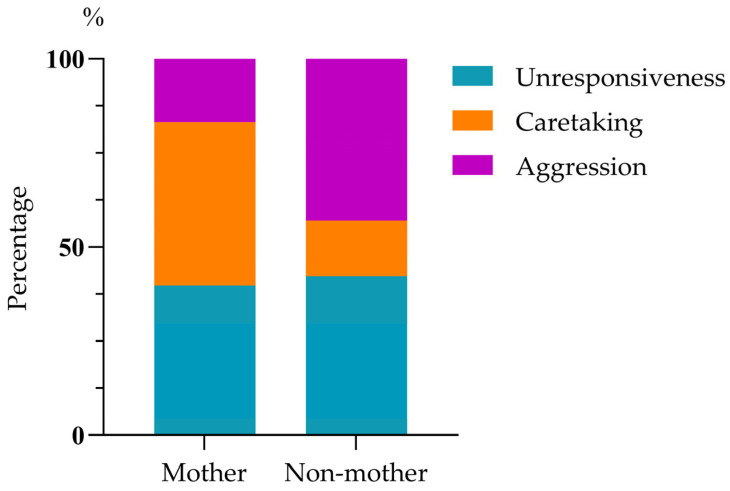
The responses of the harassed mothers and non-mothers.

Infant harassment of mating rarely led to cessation of the behavior (1.84%, 6/326). The mating male mostly ignored the harassing infant (94.17%, 307/326), rarely showed aggression (3.99%, 13/326), and even more rarely, caretaking (1.84%, 6/326). Mating females showed a greater likelihood of caretaking (31.60%, 103/326) and aggression (27.60%, 90/326) compared to males, but they were unresponsive to just over 40% of harassments (40.80%, 133/326); mating males’ and females’ responses were significantly different (chi-squared test: χ^2^(2) = 212.693, *p* < 0.01; Figure 2). Mothers responding to harassment by their infant showed unresponsiveness (39.79%, 76/191), caretaking (43.46%, 83/191), and aggression (16.75%, 32/191), a pattern significantly different from that of non-mothers in response to infant harassment of their mating: unresponsiveness (42.22%, 57/135), caretaking (14.82%, 20/135), and aggression (42.96%, 58/135) (chi-squared test: χ^2^(2) = 40.33, *p* < 0.01; Figure 3).

The GLMM revealed no overall interaction between infant sex and kinship on the frequency of the infant harasser receiving caretaking or aggression (*p* > 0.05; Table 3). Infant sex had no overall influence (*p* > 0.05; Table 3). Caretaking by mothers was a more frequent response of mothers than non-mothers to infant harassment (*p* < 0.05; Table 3). Overall, frequency of receiving care was positively correlated with frequency of infant harassment (*p* < 0.05; Table 3). By contrast, non-mothers were more frequently aggressive toward infant harassers than mothers who were harassed by their own infant (*p* < 0.05; Table 3). This frequency of receiving aggression was positively correlated with the frequency of harassment (*p* < 0.05; Table 3).

Comparing responses to harassment by mothers and non-mothers, GLMM showed no interaction between infant sex and response type (*p* > 0.05; Table 4) and no overall effect of infant sex (*p* > 0.05; Table 4). Mothers responded more often to harassment by their infant with caretaking than aggression (*p* < 0.05; Table 4) and overall frequency of mother responses was positively correlated with frequency of infant harassment (*p* < 0.05; Table 4). By contrast, the non-mothers responded to infant harassers more frequently with aggression than with caretaking (*p* < 0.05; Table 4), and frequency of responses by non-mothers was also positively correlated with frequency of infant harassment (*p* < 0.05; Table 4).

## 4. Discussion

This study investigates and describes possible underlying motivations and short-term consequences—including benefits and risks—of harassment of matings by infant *R. roxellana*. It is the first quantitative analysis of the influence of factors such as harassment behavior patterns, infant gender, and kinship with the harassment targets. Although previous analyses of harassment by juvenile and infant primates are available [7,33], they are largely qualitative and therefore do not provide concrete evidence of the effects of potentially important variables such as those mentioned above. Our quantitative analyses revealed broad behavioral similarities with infant harassment behaviors previously described in Sichuan snub-nosed monkeys and other primate species [7]. Most such harassment consists of either affiliative (e.g., gentle touching, grooming) or more agonistic behaviors (e.g., screaming, rough manipulation); it typically does not lead to the termination of the copulation.

We categorized infant harassment of matings into two types, namely “care-seeking” and “sociable”, based on their apparent intended motivations. Care-seeking harassment was especially shown by infants towards their own mother. The frequency of infant harassment was not affected by the infant’s sex but was affected by the relationship to the mating female. Specifically, infants harassed their mother’s sexual activities more frequently than those of non-mothers and targeted their mother especially with care-seeking harassment. By contrast, there was no significant difference in the frequency of sociable harassment directed at mothers and non-mothers. Although the present study does not provide direct support for the parent–offspring conflict hypothesis [7,16], which states that harassment might delay their mother’s next pregnancy [7,34], it provides quantitative evidence in line with the prediction that harassment can lead to prolonged or “extra” care for the infant, at least in the short term.

We also focused on the behavioral responses of the harassed adults for a finer-grained analysis of possible benefits and risks to infant harassers. Mating females were more responsive than males to the harassment, with mothers dispensing more care than non-mothers to the harassment. Indeed, whereas mothers very rarely responded to their infant’s harassment with aggression, non-mothers responded more aggressively, and, in fact, showed more aggression than caretaking behaviors toward harassing infants. These findings provide support for another interpretation related to parent–offspring conflict: infant harassment of their mothers’ matings is a strategy to obtain more maternal care [7,34]. However, such interference also brings the risk of punishment, although any aggressive reactions were typically mild, limited to vocal and postural threats, or grabbing. Among the Sichuan snub-nosed monkeys, non-mother females within the same OMU often exhibit allomaternal behaviors, with affiliative relationships between infants and non-mother females [35]. Therefore, an aim of infant harassment of non-mother females might be to obtain such “extra” care, but if so, it was relatively unsuccessful; as compared to mothers, non-mother females responded with a higher proportion of threats or attacks.

Another possibility is that the harassing infant simply aims to interfere with the arrival of a future competitor. Future research can help clarify why infants harass matings of non-mothers. In some species, infants excitedly harass females during mating and sometimes even harass mating males. It has been suggested that infant harassment might simply express interest in mating behavior and also that it may protect the female from aggression by the mating male [7]; however, our observations provide little support for these suggestions or that the infant is simply playing. Instead, the infant appears to be seeking an immediate social response by harassing, in particular, increased caretaking.

## 5. Conclusions

This study has limitations, especially concerning the real or long-term benefits of infant harassment of mating. Whether harassment of their mothers’ sexual activities by infant Sichuan snub-nosed monkeys can influence maternal hormone levels or actually delay impregnation remains unknown. This kind of information is necessary for directly testing the parent–offspring conflict perspective on infant harassment of matings. Infant care and lactation restrict the primate mother’s mating and delay her next pregnancy [7,15,20]. After the replacement of the resident male of a Sichuan snub-nosed monkey OMU, if an infant disappears (e.g., due to infanticide), that infant’s mother may soon mate with the new male. Reproduction is seasonal, and at the peak of the mating season, infants often appear to be more stressed. They often harass matings, although, as confirmed here, this rarely halts the mating [7]. Therefore, while it remains unknown whether infants who harass their mothers’ sexual activities obtain any long-term benefits, we have shown that they can obtain short-term benefits in the form of more care—including “extra” suckling bouts—and therefore possibly enhanced growth and survival. We plan to collect more long-term reproductive data (including interbirth intervals) to analyze whether infant harassment delays pregnancy or otherwise affects reproductive success. In parallel, measures of maternal hormone levels and physiological responses to harassment will be valuable, along with assessments of the health and growth of the infant monkeys. It could also be asked whether the frequency of harassing mothers’ matings correlates with the amount of caretaking received overall, not just in the short term; however, the answer to this question requires more intensive observations of mother-infant dyads than were conducted here.

## Figures and Tables

**Table 1:** The results of the GLMM used to determine factors influencing the frequency of infant harassment (care-seeking and sociable types).

Frequency of Infant Harassment				
Variables	*β*	S.E.	t	*p*
Intercept	5.417	0.6234	8.69	<0.001
Infant sex	0.417	0.8816	0.473	0.639
Kinship	2.417	1.0097	2.393	0.021
Infant sex × Kinship	−0.167	1.428	−0.117	0.908
**Frequency of care-seeking harassment**				
Variables	*β*	S.E.	t	*p*
Intercept	2.667	0.3518	7.581	<0.001
Infant sex	0.333	0.4975	0.67	0.506
Kinship	2.75	0.765	3.595	<0.001
Infant sex × Kinship	−0.167	1.0819	−0.154	0.878
**Frequency of sociable harassment**				
Variables	*β*	S.E.	t	*p*
Intercept	2.75	0.379	7.256	<0.001
Infant sex	0.083	0.536	0.155	0.877
Kinship	−0.333	0.5143	−0.648	0.52
Infant sex × Kinship	0	0.7273	0	1

**Table 2:** The results of GLMM used to determine factors influencing frequency of infant harassment towards their mothers towards non-mothers.

Frequency of Harassing Mother				
Variables	*β*	S.E.	t	*p*
Intercept	2.417	0.3477	6.951	<0.001
Infant sex	0.083	0.4917	0.169	0.866
Harassment type	3	0.7631	3.931	<0.001
Infant sex × Harassment type	0.083	1.0792	0.077	0.939
**Frequency of harassing non-mother**				
Variables	*β*	S.E.	t	*p*
Intercept	2.75	0.379	7.256	<0.001
Infant sex	0.083	0.536	0.155	0.877
Harassment type	−0.083	0.5171	−0.161	0.873
Infant sex × Harassment type	0.25	0.7312	0.342	0.734

**Table 3:** Results of the GLMM used to determine factors influencing the frequency of receiving caretaking and receiving aggression.

Frequency of Receiving Caretaking				
Variables	*β*	S.E.	t	*p*
Intercept	−1.025	0.2733	−3.75	<0.001
Infant sex	0.203	0.2129	0.954	0.345
Kinship	1.995	0.3298	6.049	<0.001
Infant harassment	0.312	0.0422	7.406	<0.001
Infant sex × Kinship	−0.198	0.4437	−0.446	0.658
**Frequency of receiving aggression**				
Variables	*β*	S.E.	t	*p*
Intercept	0.24	0.2553	0.94	0.352
Infant sex	0.006	0.2178	0.026	0.979
Kinship	−2.017	0.2688	−7.505	<0.001
Infant harassment	0.386	0.0376	10.269	<0.001
Infant sex × Kinship	0.064	0.3578	0.18	0.858

**Table 4:** Results of the GLMM used to determine factors influencing the frequency of responses by mothers and by unrelated females.

Frequency of the Mother Responses				
Variables	*β*	S.E.	t	*p*
Intercept	−1.24	0.376	−3.298	0.002
Infant sex	−0.025	0.3765	−0.066	0.948
Response type	1.056	0.3633	2.906	0.006
Infant sex × Response type	−0.007	0.4856	−0.014	0.989
Infant harassment	0.46	0.0491	9.364	<0.001
**Frequency of the non-mother responses**				
Variables	*β*	S.E.	t	*p*
Intercept	0.996	0.3177	3.136	0.003
Infant sex	0.064	0.2928	0.218	0.829
Response type	−2.263	0.3162	−7.157	<0.001
Infant sex × Response type	0.208	0.4206	0.494	0.624
Infant harassment	0.247	0.0445	5.541	<0.001

## Data Availability

Data are available on request.
